# Corrosion Behavior and Biocompatibility of Na2EDTA-Induced Nacre Coatings on AZ91D Alloys Prepared *via* Hydrothermal Treatment

**DOI:** 10.3389/fchem.2021.810886

**Published:** 2022-01-18

**Authors:** Meifeng He, Wenbing Lu, Dan Yu, Hao Wang, Shuai Wang, Chenggong Yuan, Aiying Chen

**Affiliations:** School of Materials Science and Engineering, University of Shanghai for Science and Technology, Shanghai, China

**Keywords:** magnesium alloy, hydrothermal method, nacre coating, corrosion resistance, cytocompatibility

## Abstract

An effective method for controlling the corrosion rate of Mg-based implants must be urgently developed to meet the requirements of clinical applications. As a naturally occurring osteoid material, nacre offers a strategy to endow biomedical Mg alloys with excellent biocompatibility, and corrosion resistance. In this study, pearl powder and NaH2PO4 were used as precursors to deposit coatings on AZ91D alloy substrates hydrothermally based on Na2EDTA-assisted induction. Na2EDTA-induced nacre coatings were fabricated at various pH values, and its chemical composition and microstructure were analyzed via energy-dispersive X-ray, scanning electron microscopy, and X-ray diffraction spectroscopy. The corrosion-resistant performance and cytocompatibility of the samples were evaluated via electrochemical measurements and *in vitro* cell experiments. Results showed that the samples hydrothermally treated under faint acid conditions present excellent corrosion resistance, whereas the samples treated under slight alkaline conditions demonstrate improved biocompatibility due to high Ca and P content and large Ca/P atomic ratio. This study provides substantial evidence of the potential value of nacre coatings in expanding the biological applications of implanted biomaterials.

## Introduction

Metallic materials have appropriate physical and mechanical properties and thus have been extensively developed in clinical practice (Dehghanghadikolaei and Fotovvati, 2019; Wang et al., 2019; Fu et al., 2020). Traditional inert metallic implants include stainless steels, titanium (Ti) alloys, and cobalt-chromium (Co-Cr) alloys, whereas biodegradable metals, such as magnesium (Mg), iron (Fe), and zinc (Zn), are applied in clinical research as a new generation of biomedical materials (Horynová et al., 2019; Su et al., 2019a; Hanas et al., 2018; Khalajabadi et al., 2016; Qin et al., 2019; Su et al., 2019b). Among these metals, Mg is the eighth-most abundant element on the surface of the earth, accounting for 1.93% of the mass of the earth’s crust, and 0.13% of the mass of the ocean (Dorozhkin, 2014; Fu et al., 2019). Mg and its alloys are lightweight, and their densities (1.74–2.0 g/cm3) are close to those of the human bones (1.8–2.1 g/cm3). Furthermore, compared with the elastic modulus of other implantable metals, that of Mg alloys (41–45 GPa) are closer to those of the human bones (3–20 GPa), thus aiding in the elimination or diminishing of the stress shielding effects at the bone/implant interface and promote new bone growth (Sezer et al., 2018; Zhang et al., 2018; Zhou et al., 2019; Öcal et al., 2020). In addition, Mg and its alloys can gradually corrode or dissolve under physiological environments, resulting in their *in vivo* biodegradability. This unique biomedical feature allows Mg-based bioresorbable implants to “disappear” over time until the damaged tissues have been regenerated, thereby avoiding the necessity for secondary surgeries (Radha and Sreekanth, 2017; Acheson et al., 2019; Li et al., 2019). Fortunately, as an essential element in metabolism (Mg^2+^ is the fourth most abundant cation in the human body) and one of the natural minerals in bone tissues, Mg not only participates in regulating various physiological functions, including stabilizing RNA and DNA and activating many enzymes, but also stimulates the growth of new bone tissues (Esmail et al., 2019; Yan et al., 2017; Kiselevsky et al., 2019; Siefen and Höck, 2019; Song et al., 2019). Moreover, the degradation products of Mg-based implants can be excreted through urine and are harmless to the human body (Ali et al., 2019).

Mg is one of the alkaline earth elements, which belong to group ⅡA of the periodic table. Alkaline earth elements tend to possess high chemical activity and easily react to oxygen in the air (Dorozhkin, 2014). Thus, when Mg is exposed to body fluids, an oxidation reaction occurs on its surface, resulting in an abrupt release of hydrogen gas, and a rapid increase in local pH (Sasikumar et al., 2019; Deepa et al., 2020; Cesarz-Andraczke et al., 2020). Unfortunately, the oxidized layers produced by this oxidation reaction are composed of MgO and Mg(OH)_2_, which are not dense enough to provide sufficient protection for Mg-based implants. Particularly, insoluble MgO and Mg(OH)_2_ are converted into soluble MgCl_2_ when damaged by chloride ions in the physiological environment, simultaneously damaging the intact plane, and aggravating the pitting corrosion of Mg (Asgari et al., 2019; Prasadh et al., 2019; Kamrani and Fleck, 2019). The exceedingly high corrosion rate of Mg-based implants in body fluids may lead to gaps at the interface of the implant and the new-formed bone. Therefore, the corrosion rate of Mg-based implants must be controlled to enhance the feasibility of bioresorbable Mg alloys for implant applications. Ideally, metallic implants should maintain their mechanical integrity during bone healing and then gradually “disappear” when the expected support is accomplished (Dorozhkin, 2014; Su et al., 2019a).

The addition of nontoxic elements with high-standard electrode potential into a Mg matrix is one of the mainstream methods for the improvement of mechanical properties and the reduction of corrosion rate of Mg alloys (Song et al., 2019; Sasikumar et al., 2019). The AZ91D alloy substrate used in this study is a typical Mg-Al-Zn alloy, which exhibits a two-phase structure consisting of an α-Mg matrix phase and a β-Mg_17_Al_12_ phase (Mena-Morcillo and Veleva, 2020; Ramalingam et al., 2020). The commercially available AZ91D magnesium alloy was investigated *in vivo* and vitro conditions and proposed to use as a matrix in magnesium composites for clinical applications (Bommala et al., 2019). For controlling the degradation rate, corrosion attack, and further dissolution of magnesium alloys, silica sol–gel coatings were deposited on AZ31B and AZ91D magnesium substrates for temporal implants (Castro and Durán, 2019).

Generally, as the main alloying element, aluminum (Al) plays a major role in refining the grain size and promoting the formation of oxidized films on the surface of the alloy that can resist local passivation (Sasikumar et al., 2019; Liu H et al., 2019). Zinc (Zn) is one of the essential nutrients for the healthy development of human body, with over 85% present in the muscles and bones. Zn can also improve the yield stress of materials and suppress the evolution of hydrogen gas in physiological conditions (Radha and Sreekanth, 2017; Song et al., 2019; Bommala et al., 2019). This alloy also contains a small amount of Manganese (Mn), which can prevent osteoporosis, and reduce the unfavorable effect of impurities (Kiselevsky et al., 2019; Hou et al., 2020).

However, adding other elements into the Mg matrix cannot satisfy the demands of clinical applications (Cui et al., 2019). Studies have shown that surface modification methods can not only delay the initial degradation of Mg and its alloys but also maintain their mechanical properties. Furthermore, the surface roughness produced by the coating can be favorable for cell adhesion and improve the biocompatibility of implants (Zhou et al., 2019; Sasikumar et al., 2019; Deepa et al., 2020). As the major inorganic constituent of natural bone, calcium phosphate (CaP) coatings, including hydroxyapatite (HA, Ca_10_(PO_4_)_6_(OH)_2_), β-tricalcium phosphate (β-TCP, β-Ca_3_(PO_4_)_2_), dicalcium phosphate dihydrate (CaHPO_4_·2H_2_O), and octacalcium phosphate (OCP, Ca_8_(HPO_4_)_2_(PO_4_)4·5H_2_O), are appropriate for moderating the biodegradation of Mg alloys, and accelerating bone concrescence (Zhang et al., 2018; Su et al., 2013). Various techniques, such as sol-gel (Su et al., 2018). Electrodeposition (Horynová et al., 2019), RF magnetron sputtering (Acheson et al., 2019), plasma electrolytic oxidation (Fu et al., 2019), chemical conversion (Duan et al., 2018; Zhang et al., 2019; Asl et al., 2020; Liu et al., 2020), and hydrothermal deposition (Tomozawa et al., 2010; Hiromoto and Tomozawa, 2011; Tomozawaa and Hiromoto, 2011; Li et al., 2012; Hiromotoa et al., 2013; Ohtsu et al., 2013; Liu P et al., 2019), have been used to deposit CaP coatings on Mg alloy substrates. Among these techniques, hydrothermal deposition is the simplest and most cost-effective because it only needs a single step of immersion (Zhang et al., 2018).

Nacre, commonly known as the mother of pearls, is a biologically formed organic-inorganic composite material, which contains 95 wt% calcium carbonate (CaCO_3_) in the form of aragonite crystal and 5 wt% complex organic matrix. The organic matrix is mainly composed of proteins and polysaccharides. Although the content of organic matrix in nacre is small, it plays a vital role in controlling the nucleation, and shape of CaCO_3_ polymorphs (Sun and Bhushan, 2012; Kwan and Wang, 2013; Gerhard et al., 2017; Raghavan et al., 2019). Nacre powder has been reported to have better biological activity and osteoinductivity compared with HA, a bioceramic widely used as bone substitute material (Wang, 2004). Nacre can be transformed into HA via reactions in phosphate buffer solutions. Calcium ions released from the nacre surface or nacre powder can interact with free phosphate within the phosphate buffer solutions and then precipitate as HA (Ni and Ratner, 2003; Wu et al., 2011; He et al., 2016). Moreover, the organic matrix of nacre plays a critical role in HA particle formation (Kobayashi et al., 2012).

Consequently, a nacre coating was hydrothermally deposited on AZ91D alloy substrates using pearl powder and NaH_2_PO_4_ as precursors on the basis of Na_2_EDTA-assisted induction. This coating can promote the cytocompatibility and corrosion resistance of Mg alloys simultaneously. In addition, the chemical composition, micromorphology, corrosion resistance, and cytocompatibility of the coatings were analyzed comprehensively.

## Experimental Section

### Sample Preparation

Mg alloy substrates must be pretreated to obtain coatings with strong binding force and high purity. The AZ91D bar was cut into 10 × 10 × 3 mm samples. Each sample was ground to 800 #, 1,200 #, and 1,500 # with SiC papers. After cleaning ultrasonically in ethanol, samples were chemically polished with 8% HNO_3_ + 1% H_2_SO_4_ (vol%) solution. Chemically polished samples were cleaned ultrasonically in ethanol again, followed by air drying.

The solution was prepared with Na_2_EDTA, pearl powder, and NaH_2_PO_4_ with a concentration of 0.5 mol/L. Sodium hydroxide was used to adjust PH values to 5.5, 8.5, and 11.5, respectively. The samples were immersed in 50 ml hydrothermal reactors solutions of 5.5, 8.5, and 11.5 pH values at room temperature. Subsequently, the treatment solutions were heated to 363 K and then maintained for 6 h.

### Surface Characterization

The phase composition of the coatings was analyzed via X-ray diffraction (XRD), whereas with help of scanning electron microscopy (SEM) and Energy-dispersive X-ray spectroscopy (EDX), the surface morphology, and the chemical composition of the coatings were analyzed.

### Electrochemical Measurement

In a simulated body fluid (SBF), an electrochemical measurement system was used to evaluate the corrosion resistance of samples. The chemical composition of the SBF solution (1 L) is shown in Table 1. A classical three-electrode setup was used for electrochemical measurements; in this setup, a platinum sheet, a saturated calomel electrode (SCE), and the tested samples act as counter, reference, and working electrodes, respectively. First, the sample was immersed in SBF for 1,200 s to acquire a stable open circuit potential (OCP). Second, electrochemical impedance spectroscopy (EIS) was performed at OCP values from 100 to 0.01 Hz. Third, with a scanning rate of 5 mV/s, the potentiodynamic polarization curve of the samples was measured.

**TABLE 1:** The chemical composition of SBF solution (g).

NaCl	KCl	CaCl_2_	NaHCO_3_	MgCl_2_	NaH_2_PO_4_	Glucose
18.00	0.20	0.20	1.00	0.10	0.05	1.00

### Cytocompatibility Test

Human SV40 transfected osteoblasts were cultured and passaged in a D-MEM/F-12 medium containing 10% fetal bovine serum, and the CCK-8 method was used to assess the effects of the sample extracts on cell growth. In general, the cells were plated as needed in 96-well plates (100 μl/well and 1 × 10^5^ cells/well) and then were placed in an incubator for (37°C, 5% CO_2_) 1 day to promote the complete adherence procedure. Next, the above culture medium was replaced with a fresh culture medium containing the sample extracts for further cultivation. After various durations (24, 48, and 72 h), the D-MEM/F-12 medium containing 10% (v/v) CCK-8 (100 μl/well) was used to incubate the cells for final measurement on optical density (OD) values using a microplate reader. A proportional relationship exists between the OD values and the numbers of the cells; therefore, the OD values can reflect the viability of the cells. Herein, the cellular activity of the negative control group was set to unit 1, and then the cellular activity of the other groups could be calculated using the following formula:

$$\text{RGB\%}=\left[\frac{\text{OD}\left(\text{sample}\right)-\text{OD}\left(\text{positive}\right)}{\text{OD}\left(\text{negative}\right)-\text{OD}\left(\text{positive}\right)}\right]\times 100\text{\%}\text{.}$$ (1)

**FIGURE 1:**
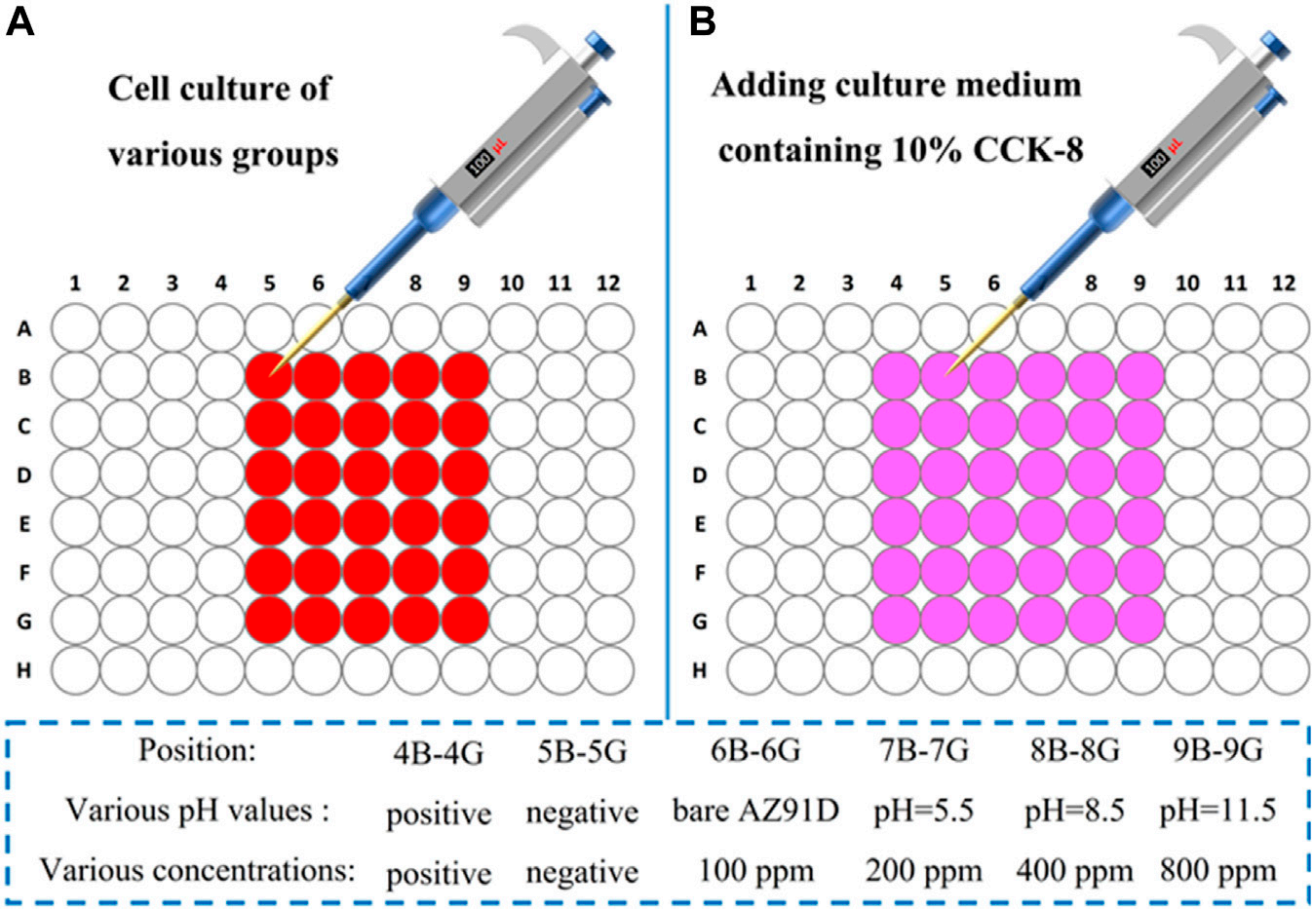
Instruction of cytotoxicity tests.

In this section, OD (sample) represents the OD values of the cells incubated in the medium containing the sample extracts, OD (positive) represents the OD values of the D-MEM/F-12 medium containing 10% (v/v) CCK-8, and OD (negative) represents the OD values of the cells incubated in the normal medium. The associated instruction of cytotoxicity tests is shown in Figure 1 **.**

The cytocompatibility of samples was further investigated using the confocal microscopic imaging of the cells. Similarly, the cells were first plated in the culture dishes (1 ml/well and 1 × 10^6^ cells/well) and then were placed in an incubator for (37°C, 5% CO_2_) 1 day to promote complete adherence. Next, the fresh culture medium containing the sample extracts was used to replace the previous medium for further cultivation. After 24, 48, and 72 h, the above culture medium was removed, the cells were washed with PBS solution thrice, and the PBS solution was added with calcein-AM/PI (a living cell/dead cell double staining kit) into the culture dishes for cellular staining (15 min), respectively. Then, cells were washed with the PBS solution (thrice) to remove the excess fluorescent dye. Lastly, the stained cells were immersed in 100 μl of PBS solution for subsequent observation via confocal laser scanning microscopy. The living/dead cells can be observed with green/red fluorescence.

## Results and Discussion

### Composition and Morphology of the Coatings

**FIGURE 2:**
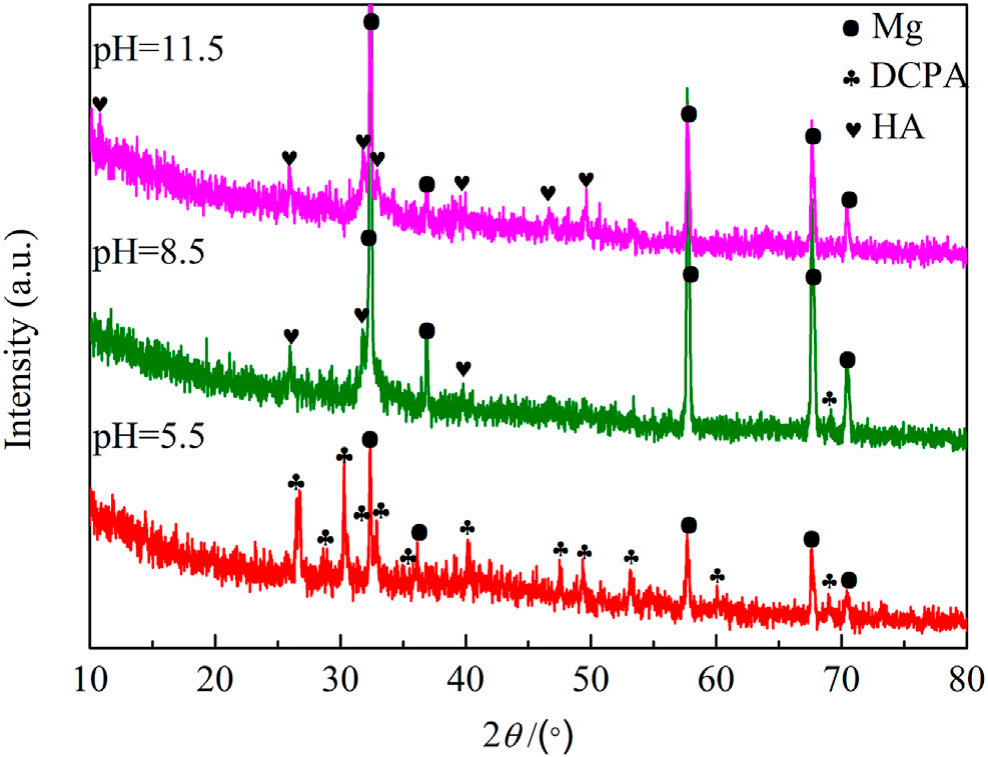
XRD patterns of samples treated at different pH values.

Figure 2 shows the XRD patterns of the samples treated in the hydrothermal solution at various pH values. On the sample of pH 5.5-treated, CaHPO_4_ (DCPA) diffraction peaks are observed apart from the peaks from the AZ91D substrate. On the sample of pH 8.5-treated, HA-rich peaks with small peaks from DCPA are observed apart from the peaks of the Mg substrate. On the sample of pH 11.5-treated, the DCPA peaks disappear, and only the HA peaks but the substrate peaks are observed. Simultaneously, the Mg peaks of the pH 5.5-treated AZ91D are far lower than those of the others. The crystallinity of the coatings on the pH 5.5-treated samples is higher.

**FIGURE 3:**
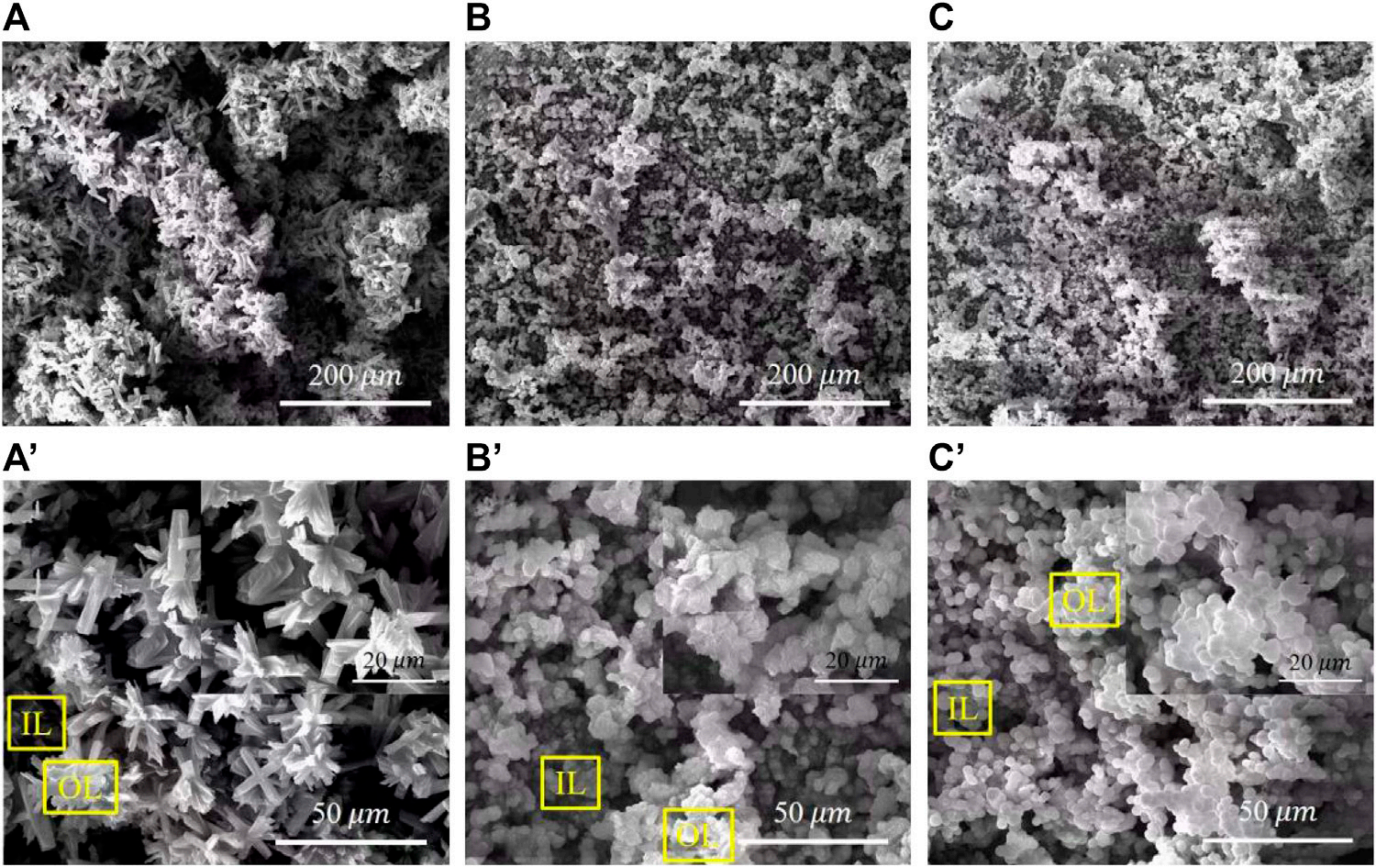
SEM images of various pH values treated samples with different magnification: **(A,A')** the pH 5.5-treated, **(B,B')** the pH 8.5-treated, and **(C,C')** the pH 11.5-treated.

Figure 3 shows the surface morphologies of various PH values treated samples. The deposited coatings densely cover the surface with agglomerates in various morphologies. All coatings have a layered structure, confirming that the coating crystals continuously precipitate on the surface and grow layer by layer. Figure 3A shows that the surface of the pH 5.5-treated samples is covered with flower-like aggregates of approximately 15 μm in diameter. Lamellar crystals are in varying lengths and interlaced together at a certain angle in each flower-like structure. On the sample of pH 8.5-treated, flocculent precipitates of approximately 7 μm were uniformly and densely covered on the surface. Observed at a higher magnification, and the flocculent precipitates comprise fine flakes (Figure 3B). The surface morphology at pH 11.5 is similar to the pH 8.5-treated AZ91D, but the crystal size of the coatings tends to be much finer and more uniform (Figure 3C).

**FIGURE 4:**
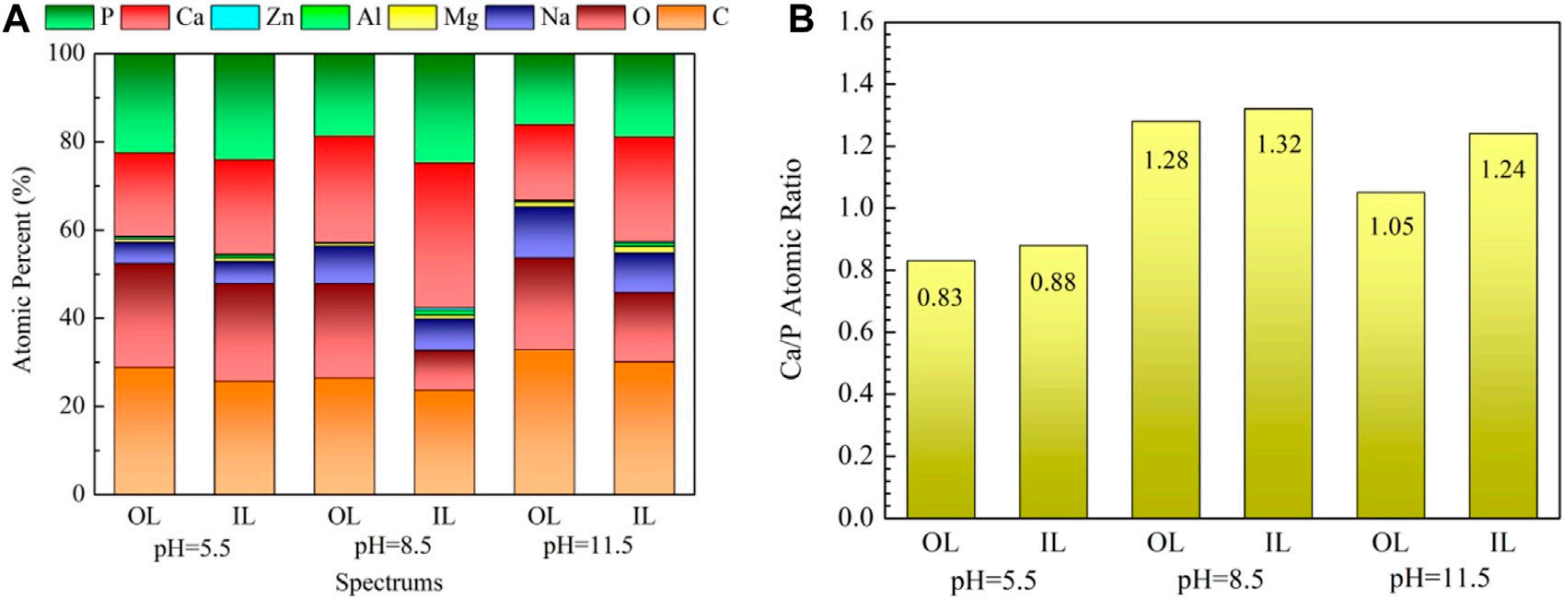
EDX results of the marked spots in Figure 3: **(A)** EDX spectra, **(B)** Ca/P atomic ratio.

The surface composition of the coated samples acquired via EDX is shown in Figure 4 **.** All deposited coatings are rich in Ca, P, O, and C and contain almost no Mg, indicating that coatings have completely covered the surface of the substrates. Given that the α-Mg matrix is more prone to corrosion in a high alkaline hydrothermal environment, the sample treated at pH 11.5 contains more Mg compared with the others. The Ca/P atomic ratio of the OL spot (outer layer) on the samples treated at pH 5.5, 8.5, and 11.5 is 0.83, 1.28, and 1.05, whereas that of the IL spot (inner layer) is 0.88, 1.32, and 1.24, respectively. The fact that the Ca/P atomic ratio of the inner layer is higher than that of the outer layer may be due to the decrease in the Ca and P content in the solution at the later period of the hydrothermal reaction. The Ca/P atomic ratio obtained in this study is slightly lower than the theoretical value because the Na and Mg in the hydrothermal solution replace the Ca in the nacre coatings (Zhang et al., 2018).

### Electrochemical Behavior of Bare and Coated AZ91D Alloys

**FIGURE 5:**
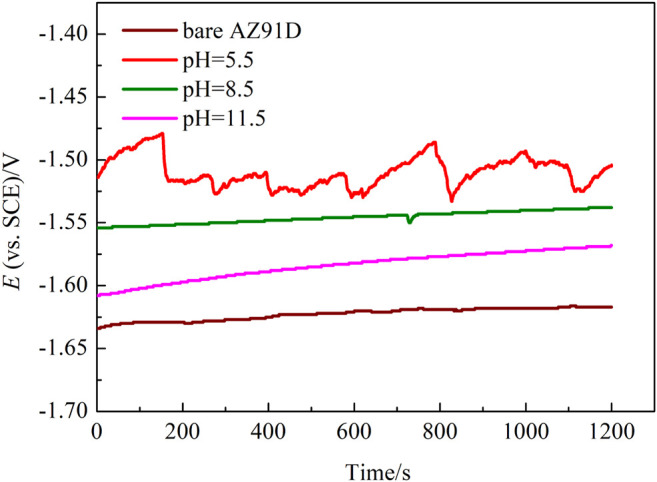
Open circuit potential curves of bare AZ91D and pH–treated samples in SBF.

Figure 5 shows the OCP of bare AZ91D and the pH–treated samples in SBF within 1,200 s. In general, the pH–treated samples reveal more inert potential than the bare AZ91D alloy (−1,617 mV/SCE). The hydrothermally treated sample at pH 5.5 shows the highest OCP value, with a relatively stable potential value of approximately −1,505 mV/SCE. Compared with the OCP curve of the other samples, that of the pH 5.5-treated AZ91D fluctuates irregularly, indicating that the uniformity of the coating is low. The OCP of pH 8.5-treated AZ91D is second only to that of the pH 5.5-treated AZ91D, with a potential that stabilizes at approximately −1,538 mV/SCE. The OCP of the pH 11.5-treated AZ91D varies from a lower potential (−1,608 mV/SCE) to a relatively stable higher potential (−1,568 mV/SCE), as a result of the coating drawbacks that expose the Mg substrate to electrolytes at the beginning of the test. The corrosion products that accumulate in the pores and cracks of the coating limit the further corrosion of the substrate, resulting in the increase in OCP to the positive direction (Jayaraj et al., 2016).

**FIGURE 6:**
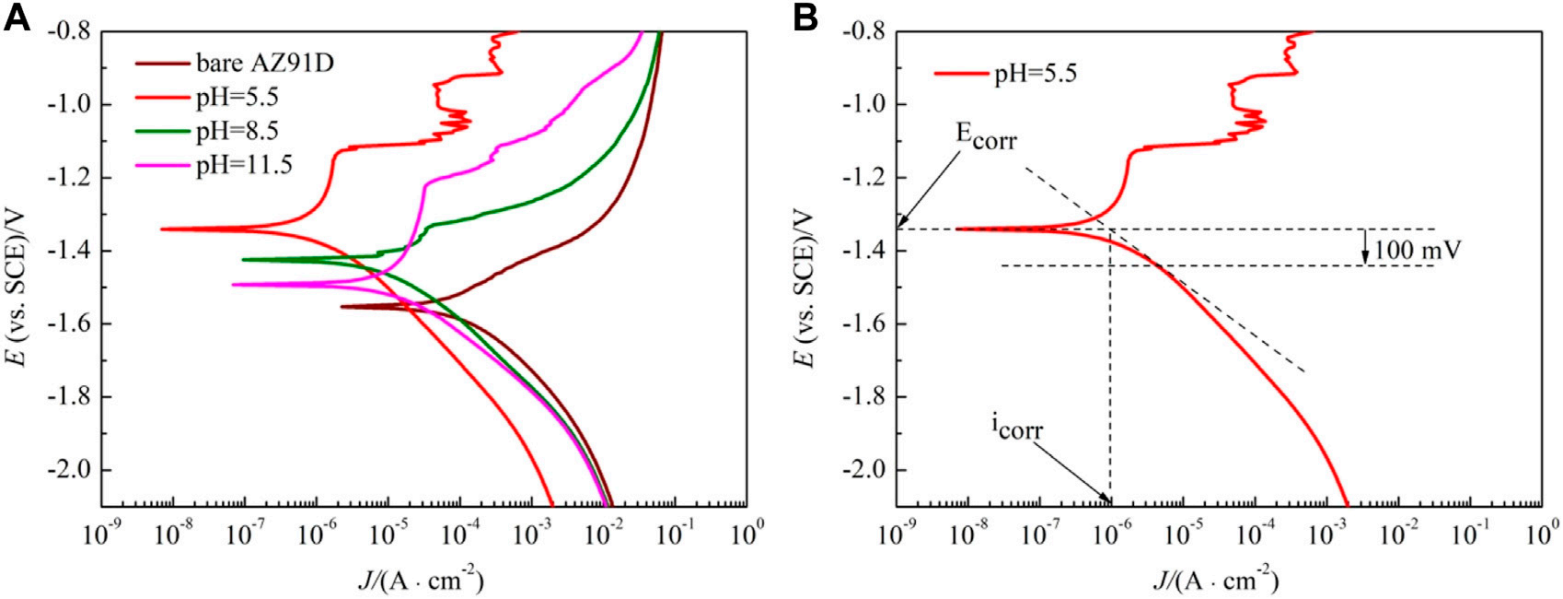
Polarization curves of bare AZ91D and pH–treated samples in SBF: **(A)** polarization curves, **(B)** diagram of curve fitting.

The polarization curves of the samples are shown in Figure 6A. The anodic polarization curve of bare AZ91D appears active dissolution, whereas the coated samples generate multiple passivation behavior. Table 2 presents the corresponding electrochemical data obtained from the polarization curves. Given the abnormal behavior of Mg samples during anodic dissolution, the cathodic polarization zone is only used for estimating corrosion current density (i_corr_) (ref. Figure 6B) (Kobayashi et al., 2012). The corrosion potential (E_corr_) and breakdown potential (E_break_) of coated samples are much higher than those of bare AZ91D, indicating that the nacre coatings give protection to the substrate. In addition, the Tafel curves of the coated samples shift toward the left side to that of bare AZ91D, resulting in reduced i_corr_ for coated samples. Specifically, the decrease in i_corr_ for the hydrothermally treated sample at pH 5.5 is more predominant than that for samples treated at pH 8.5 and 11.5, which may be caused by the thick coating after the pH 5.5-treated.

**TABLE 2:** Fitted results of polarization curves in Figure 6A.

Samples	E_corr_ (V/SCE)	i_corr_ (μA/cm^2^)
bare Z91D	−1.56	104
pH = 5.5	−1.34	0.961
pH = 8.5	−1.43	9.01
pH = 11.5	−1.49	14.1

**FIGURE 7:**
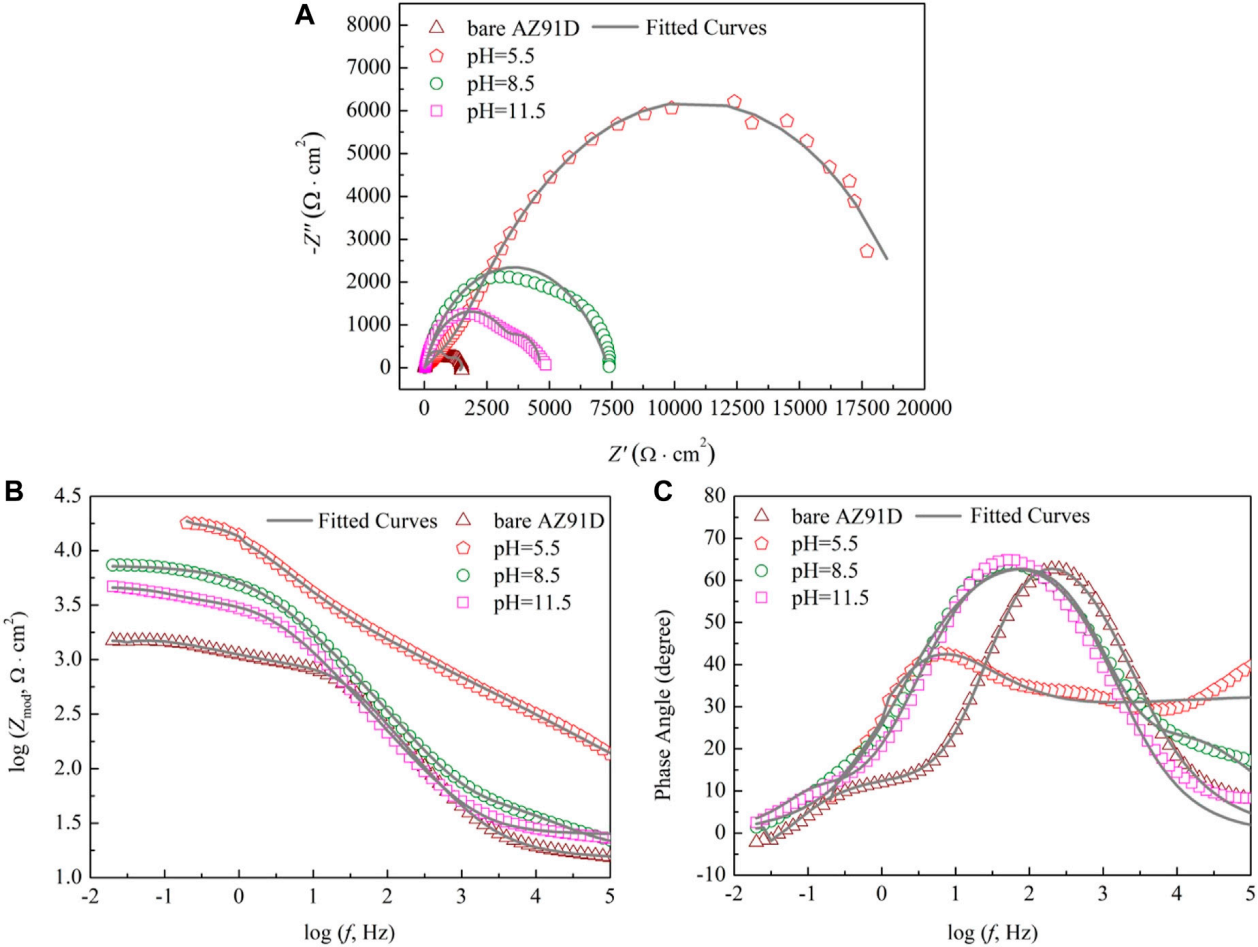
Impendence spectra of bare AZ91D and coated samples: **(A)** Nyquist plots, **(B)** Bode plots of log *Z*_mod_ vs log *f* and **(C)** Bode plots of phase angle vs log *f*.

To understand the degradation mechanism of bare AZ91D and coated samples in SBF, EIS plots are used to evaluate the corrosion resistance of samples. Figure 7A shows the Nyquist plots of the bare and coated samples. The bare sample exhibits capacitive loops at high and medium frequencies and an inductive loop at low frequencies, whereas the coated samples only show two capacitive loops at high and medium frequencies. Generally, for bare AZ91D, the capacitive loop at high frequencies represents that hydroxides and oxides are generated at the metal-solution interface due to the corrosion that occurs in the electrochemical test. Equally, for samples after the pH-treated, the presence of a protective film on the substrate affects the capacitance loop at high frequencies. The capacitance loop at medium frequencies denotes the region of the mass transfer in the solid phase as a result of the diffusion of Mg^2+^ through oxides, hydroxides, or phosphates on pH–treated (coated) samples on the exposed AZ91D. The presence of the inductive loop attributed to the adsorption-desorption of ions suggests that pitting corrosion has occurred on the surface of bare AZ91D (Jayaraj et al., 2016; Zhang et al., 2018).

**FIGURE 8:**
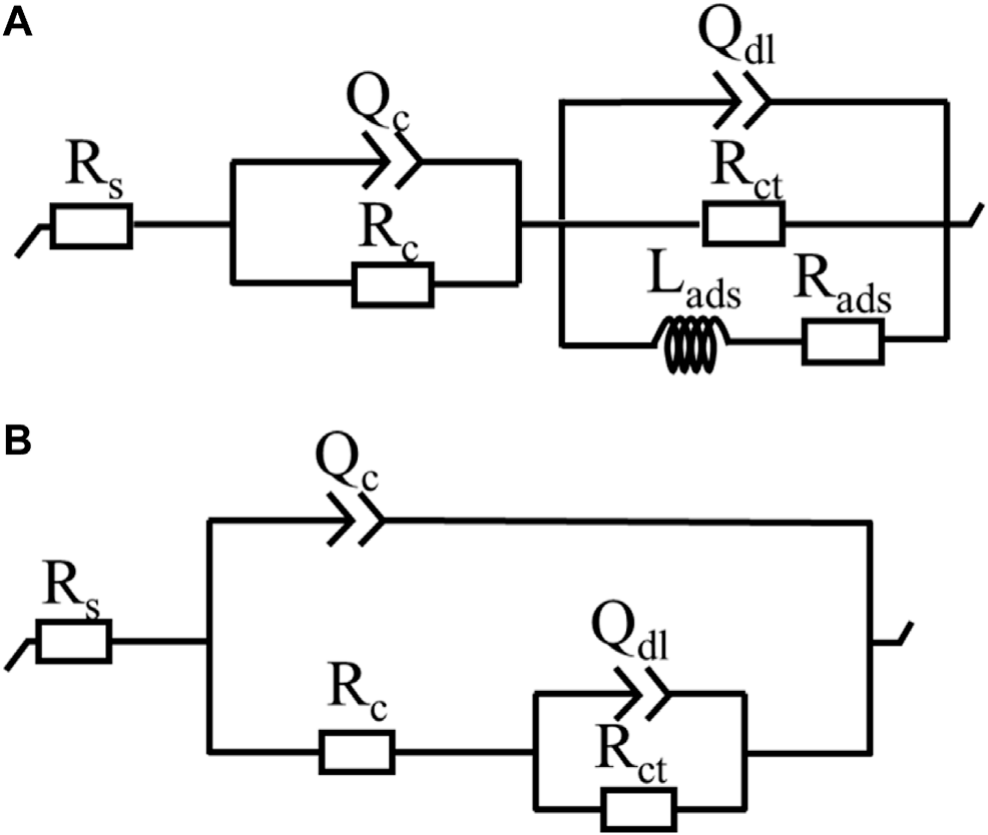
Equivalent circuits for EIS plots: **(A)** bare AZ91D, **(B)** coated samples.

The results of the electrochemical measurement are shown in Bode plots of log *Z*_mod_ vs log *f* (Figure 7B) and phase angle vs log *f* (Figure 7C) too. Compared with the *Z*_mod_ of bare AZ91D, that of the coated samples increases at low frequencies, indicating that the coatings can decelerate the dissolution of substrates during electrochemical corrosion. Similarly, the Bode phase angle plot shows that coated samples have high phase angles (toward 70°), revealing that the nacre coatings effectively provide corrosion protection for substrates (Kobayashi et al., 2012). The EIS data of bare AZ91D and coated samples can be fitted with two kinds of equivalent electrical circuits, as shown in Figure 8 **.** R_s_ represents the solution resistance. R_c_ and Q_c_ connected in parallel denote the capacitive loop at high frequencies relevant to the dielectric properties of the coating. R_ct_ and Q_dl_ in parallel describe the capacitance loop at medium frequencies. R_ct_ represents the charge transfer resistance, and Q_dl_ is a double-layer capacitor at the interface of the solution and substrate. Q stands for constant phase element and is represented by *Y = Y*_*0*_ *(jω)* ^*n*^, where *Y*_*0*_ is admittance, *n* is the power index number (ref. Table 3, *n*_*1*_ for coating, *n*_*2*_ for double layer), *j* is an imaginary unit, and *ω* is the angular frequency. To obtain an improved quality of fit, it is frequently applied to replace the capacitor (Jayaraj et al., 2016; Maurya et al., 2017). R_ads_ and L_ads_ represent the inductive loop at low frequencies and are normally related to the exposure of the Mg substrate and the release of Mg^2+^ and Mg(OH)_2_ (Cui et al., 2019).

**TABLE 3:** The fitted values of EIS plots for Figure 7.

	Bare AZ91D	pH 5.5-treated	pH 8.5-treated	pH 11.5-treated
R_s_ (Ω cm^2^)	14.77	28.99	16.76	25.04
Q_c_ = Y_0_ (Ω^−1^ cm^−2^ s^n1^)	7.09 × 10^−4^	5.88 × 10^−6^	2.10 × 10^−5^	2.76 × 10^−5^
n_1_	0.53	0.54	0.65	0.79
R_c_ (Ω·cm^2^)	1,171	844.7	50.45	3,643
Q_dl_ = Y_0_ (Ω^−1^ cm^−2^ s^n2^)	1.21 × 10^−5^	1.17 × 10^−6^	5.23 × 10^−6^	1.15 × 10^−5^
n_2_	0.92	0.62	0.89	0.96
R_ct_ (Ω cm^2^)	746.4	21,600	7,297	986.2
L_ads_ (H/cm^2^)	6,793	—	—	—
R_ads_ (Ω cm^2^)	486.1	—	—	—

The fitted results of each component utilized in the equivalent circuit are summarized in Table 3. R_s_ of coated samples is higher than that of bare AZ91D, suggesting that the coating can effectively delay the penetration of electrolyte into the substrate. Generally, a high R_ct_ means improved corrosion resistance. R_ct_ of samples increases in the following order: uncoated < pH 11.5-treated < pH 8.5-treated < pH 5.5-treated. Likewise, a low Q_dl_ implies that corrosion has occurred on a relatively minor area. Q_dl_ of coated samples is lower than that of bare AZ91D, confirming that the nacre coating has better protection compared with the corrosion-oxidized layer formed on the surface of bare AZ91D. The values of n_2_ are all higher than that of n_1_, indicating that the rough oxidized layer (on bare AZ91D), and the nacre coatings (on coated samples) become smooth during the electrochemical reaction. The sum of R_c_ and R_ct_ is the polarization resistance (R_p_), which is proportional to the diameter of the capacitive loop (Zhang et al., 2018). Therefore, R_p_ of the coated samples is larger than that of bare AZ91D (1917.4 Ω cm^2^), and the AZ91D has the largest R_p_ (22,444.7 Ω cm^2^) after treatment at pH = 5.5; these results are consistent with the Nyquist plots (ref. Figure 7A).

**FIGURE 9:**
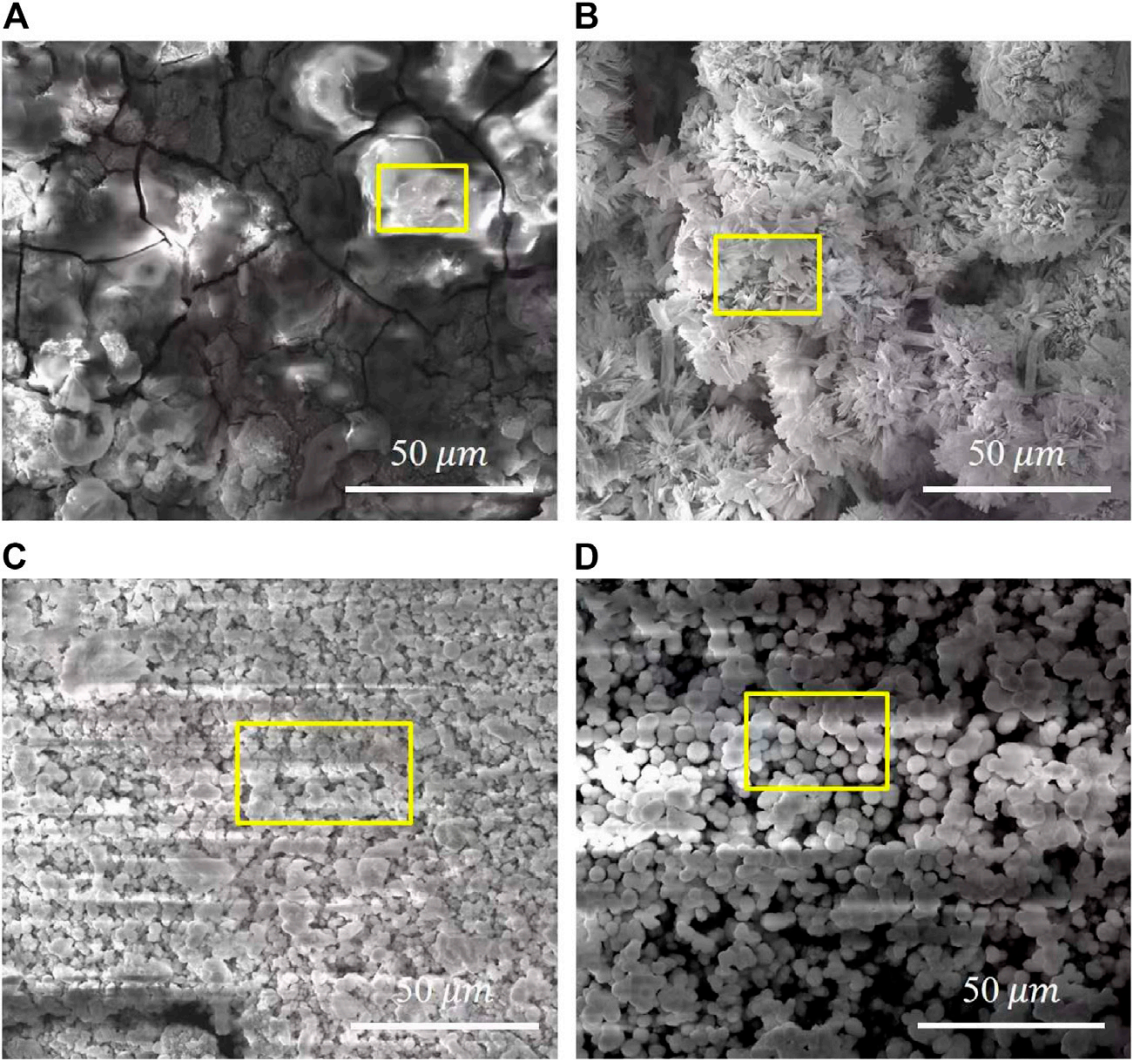
Surface morphology of samples after electrochemical measurement: **(A)** bare AZ91D, **(B)** pH 5.5-treated, **(C)** pH 8.5-treated, **(D)** pH 11.5-treated.

**FIGURE 10:**
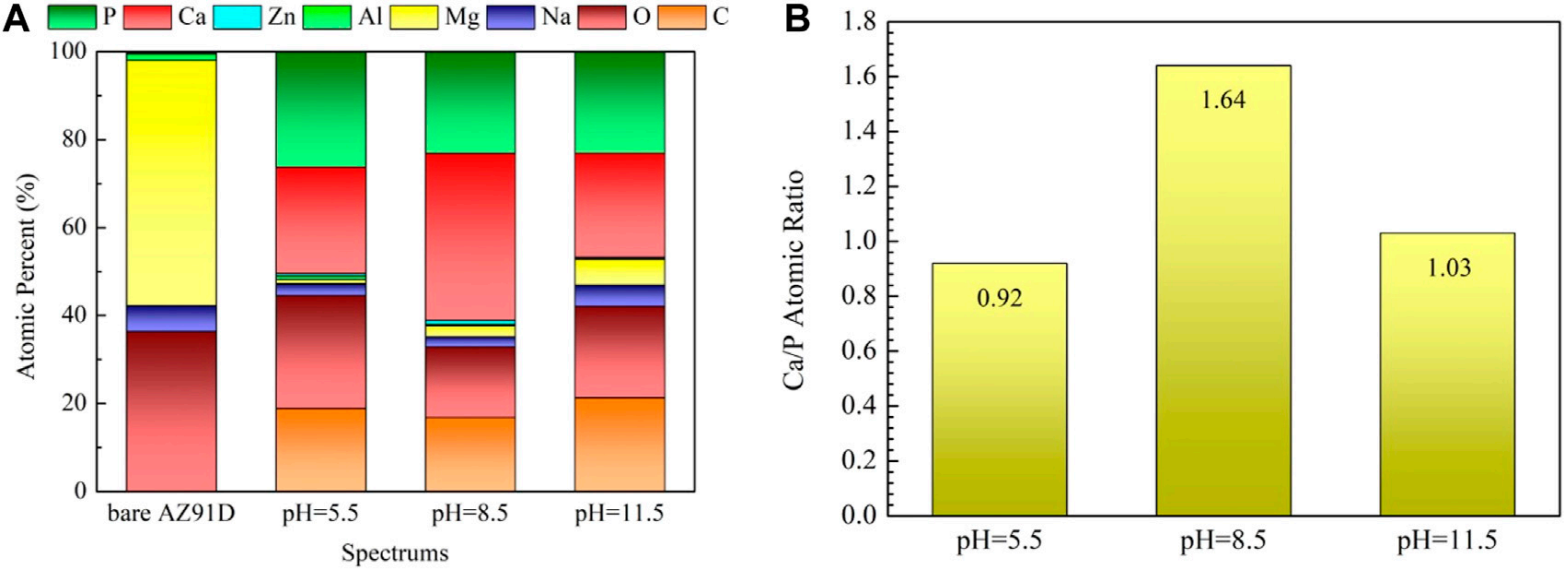
EDX results of the areas marked in Figure 9: **(A)** EDX spectra, **(B)** Ca/P atomic ratio.

Figure 9 shows the SEM micrographs of samples after electrochemical measurement in SBF. The chemical composition of the corresponding sections in Figure 9 acquired via EDX are shown in Figure 10. The bare AZ91D degrades faster in SBF; moreover, large areas of corrosion products and numerous cracks are found on its surface. The corresponding EDX results show that the bare AZ91D surface mainly contains Mg and O with a small amount of Na and Al but does not contain any Ca and P. These results confirm that the corrosion products on bare AZ91D are mainly composed of Mg(OH)_2_. By contrast, the surface of the coated samples is almost unaffected, but the morphology of the coatings is slightly changed. This result is probably due to the slight dissolution of coating particles in the early stage of electrochemical corrosion and the further deposition of corrosion products on the remaining coating surface or in the micropores of the coating as the reaction occurs. The morphology of corrosion products on the pH 5.5-treated AZ91D is similar to that of dense dandelion-like aggregates, which are rich in Ca, P, O, and C with a Ca/P atomic ratio of 0.92, and is almost free of alloy elements of the substrate, such as Mg, Al, and Zn. On the pH 8.5-treated AZ91D, the shapeless aggregates mainly containing Ca, P, O, and C with a small amount of Mg cover the surface densely, with a Ca/P atomic ratio of 1.64, which is close to the theoretical Ca/P atomic ratio of HA (1.67) (Su et al., 2019a; Dorozhkin, 2014). On the pH 11.5-treated AZ91D, corrosion products are sparsely deposited on the surface in a granular form, with a Ca/P atomic ratio of 1.03. The Mg content is slightly high due to the presence of pores. The Mg content of samples increases in the following order: pH 5.5 (0.95 at%) < pH 8.5 (2.54 at%) < pH 11.5 (5.91 at%) < uncoated (55.77 at%). The above rule is consistent with the results of electrochemical tests, further confirming that the nacre coating can effectively prevent the penetration of the solution and inhibit the corrosion of the Mg alloy substrate. Moreover, the Ca and P content on the surface of all coated samples and the Ca/P atomic ratio of samples treated at pH 5.5 and 8.5 increase after the electrochemical test (ref. Figure 4) probably because when the nacre coating is immersed in SBF, the ion exchange controls its dissolution, and electrostatic interactions between negatively charged groups on the surface and ions in SBF trigger further biomineralization (Maurya et al., 2017).

**FIGURE 11:**
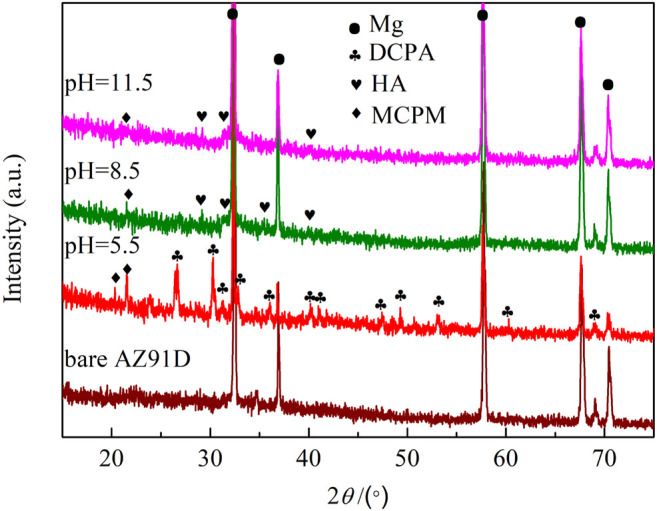
XRD patterns of samples after electrochemical measurement.

The XRD patterns of samples after electrochemical measurement in SBF are given in Figure 11. On the pH 5.5-treated AZ91D, Apart from the peaks from Mg DCPA-rich peaks with small peaks from Ca(H_2_PO_4_)_2_·H_2_O (MCPM) are observed, indicating that the surface of the sample treated at pH 5.5 is completely preserved, and a small number of corrosion products are generated. However, the HA peaks of the samples treated at pH 8.5 and 11.5 weaken, revealing that the coatings are slightly corroded. Thus, the above results further confirm that the sample treated at pH 5.5 has the best corrosion resistance.

### Cytocompatibility of Bare and Coated AZ91D Alloys

**FIGURE 12:**
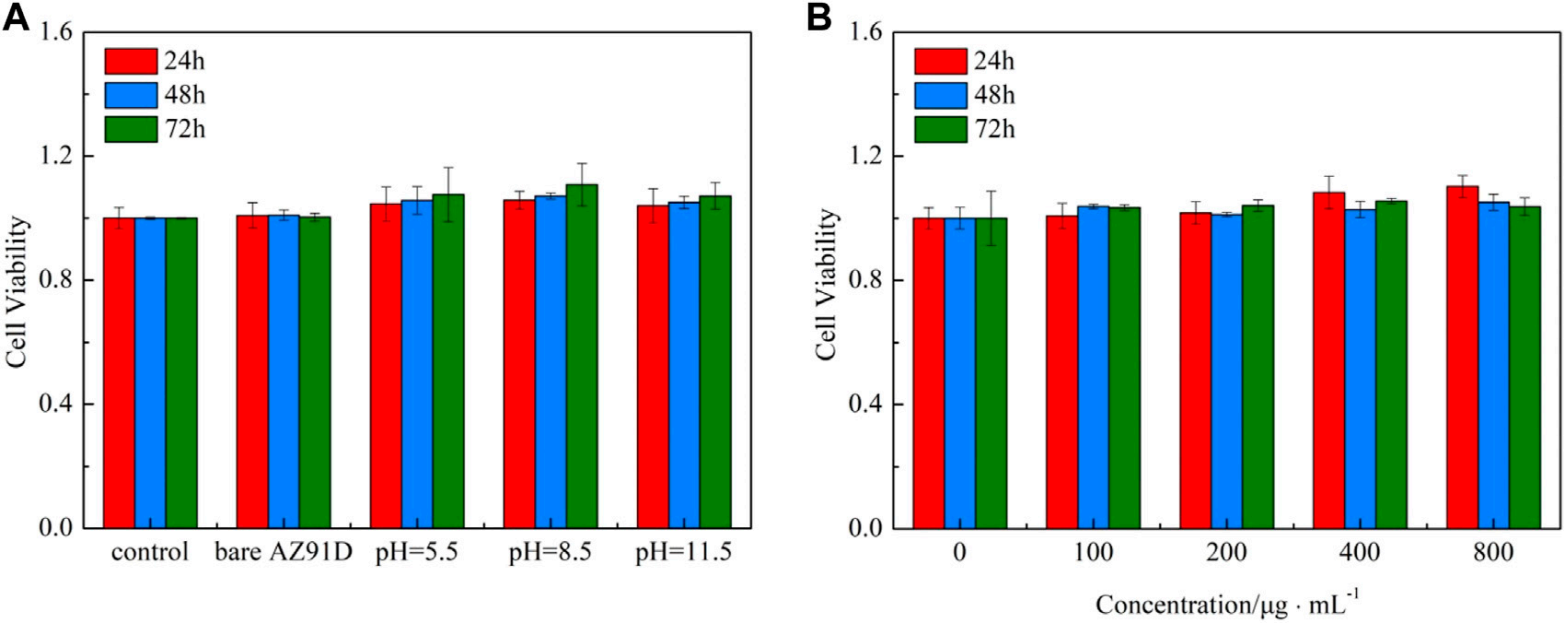
CCK-8 cytotoxicity test: **(A)** Viabilities of 24, 48, and 72 h for human SV40 transfected osteoblasts in various groups, **(B)** Viabilities of 24, 48, and 72 h for human SV40 transfected osteoblasts in the pH = 8.8 group.

The cytotoxicity test is crucial for evaluating the biocompatibility of biomaterials. Herein, the CCK-8 method is used to assess the effects of the material extracts on cellular viability. The viabilities of 24, 48, and 72 h for human SV40-transfected osteoblasts after being incubated in the extract of AZ91D alloy and the samples of hydrothermal treatment at different pH values are shown in Figure 12A. The AZ91D alloy features relatively great cytocompatibility and is thus used as the substrate. However, the cellular viability of the AZ91D alloy group decreases with the extension of the culture time. The reason may be that the excessive degradation of AZ91D alloy during the preparation of extracts causes the high pH value of the extract solution, which is not conducive to cell growth. Compared with the AZ91D alloy group, the cellular viabilities of the coated sample groups increase with the extension of culture time and are considerably higher than that of the AZ91D alloy group at each time point. This result indicates that the osteocytes maintain a good tolerance to nacre coatings. The pH 8.5 group shows higher cellular viability compared with that of the pH 5.5 and 11.5 groups probably because of the high Ca and P content in the sample treated at pH 8.5. In addition, the viabilities of 24, 48, and 72 h for human SV40-transfected osteoblasts in the pH 8.5 group are further tested. As shown in Figure 12B, the cellular viabilities exhibit an increasing trend with the extension of culture time when the concentration of the extract is low. When the concentration of the extract is high, the cellular viabilities considerably increase after cocultivation for 24 h and decrease slightly for the following days but are still higher than that of the control group. In conclusion, the above results confirm that the degradation products of nacre coatings can promote cell growth and further improve the cytocompatibility of AZ91D alloys.

**FIGURE 13:**
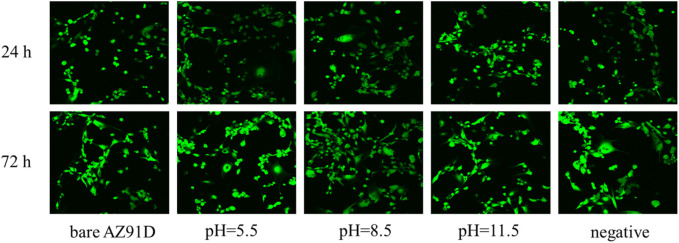
Confocal fluorescence images of human SV40 transfected osteoblasts after co-incubation for 24 and 72 h in various groups.

Furthermore, the confocal microscopic images of cells containing AM/PI fluorescent dyes are used to demonstrate the cell growth of various groups vividly (Figure 13). All cells show green fluorescence, and no red fluorescence can be found, indicating that the extract of the AZ91D alloy or various coatings has no toxic effect on the cells. After coincubation for 24 h, no considerable difference in cell number and morphology can be found between the bare AZ91D group and the negative control group. Comparatively, the cells of the other three groups that deposited with nacre coatings exhibit greater density distribution, better ductility, and more pseudopodia. After coincubation for 72 h, the cell density considerably increases, and the cell number of the coating groups is more than that of the bare AZ91D group and the negative control group, indicating that the nacre coatings have good cytocompatibility. The pH 8.5 group shows the largest cell density, covering almost the entire field of vision. The results of confocal microscopic images are consistent with that of cytotoxicity tests, thus confirming that the nacre coating can further improve the biocompatibility of AZ91D alloys and induce the nucleation and proliferation of osteoblasts.

## Mechanism Discussion

### Deposition Mechanism of Coatings

The acid pretreatment process enhances the surface energy of AZ91D alloy substrates and supplies numerous active sites. Soon after immersion in the hydrothermal solution, a passive Mg(OH)_2_ layer is formed on the substrate surface. EDTA acts as an intermediate bridge, which can not only promote the dissolution of pearl powder but also induce Ca^2+^ in the hydrothermal solution to precipitate on the substrate. Furthermore, under the influence of thermal convection and concentration gradient of the hydrothermal system, the ions, molecules, and or ionic groups in the hydrothermal solution, as well as the active ingredients in the pearl powder, are continuously delivered to the growing region. The following major reactions occur during the formation of DCPA and HA coatings:

First, Mg dissolves on the surface of the AZ91D substrate in the hydrothermal environment and reacts with H_2_O to produce Mg(OH)_2_ and H_2_.

$$Mg+2H_{2}O\to Mg^{2+}+2OH^{-}+H_{2}↑$$ (2)

The concentration of hydroxide ions on the substrate is increased by the rapid degradation of Mg and then the pH value around the alloy raises sharply. The OH^−^reacts with phosphate anions as follows:

$$H_{2}PO_{4}^{-}+OH^{-}\to HPO_{4}^{2-}+H_{2}O,$$ (3)

$$H_{2}PO_{4}^{-}+2OH^{-}\to PO_{4}^{3-}+2H_{2}O.$$ (4)

The adsorption of HPO_4_^2−^ and PO_4_^3−^ provides a negatively electrical substrate surface and promotes the primary nucleation of nacre coating.

$$Ca^{2+}+HPO_{4}^{2-}\to CaHPO_{4}\;\left(pH=5.5\right)$$ (5)

$$10Ca^{2+}+6PO_{4}^{3-}+2OH^{-}\to Ca_{10}{\left(PO_{4}\right)}_{6}{\left(OH\right)}_{2}\;\left(pH=8.5\;\&\;pH=11.5\right)$$ (6)

### The Degradation Control Mechanism of Coatings

**FIGURE 14:**
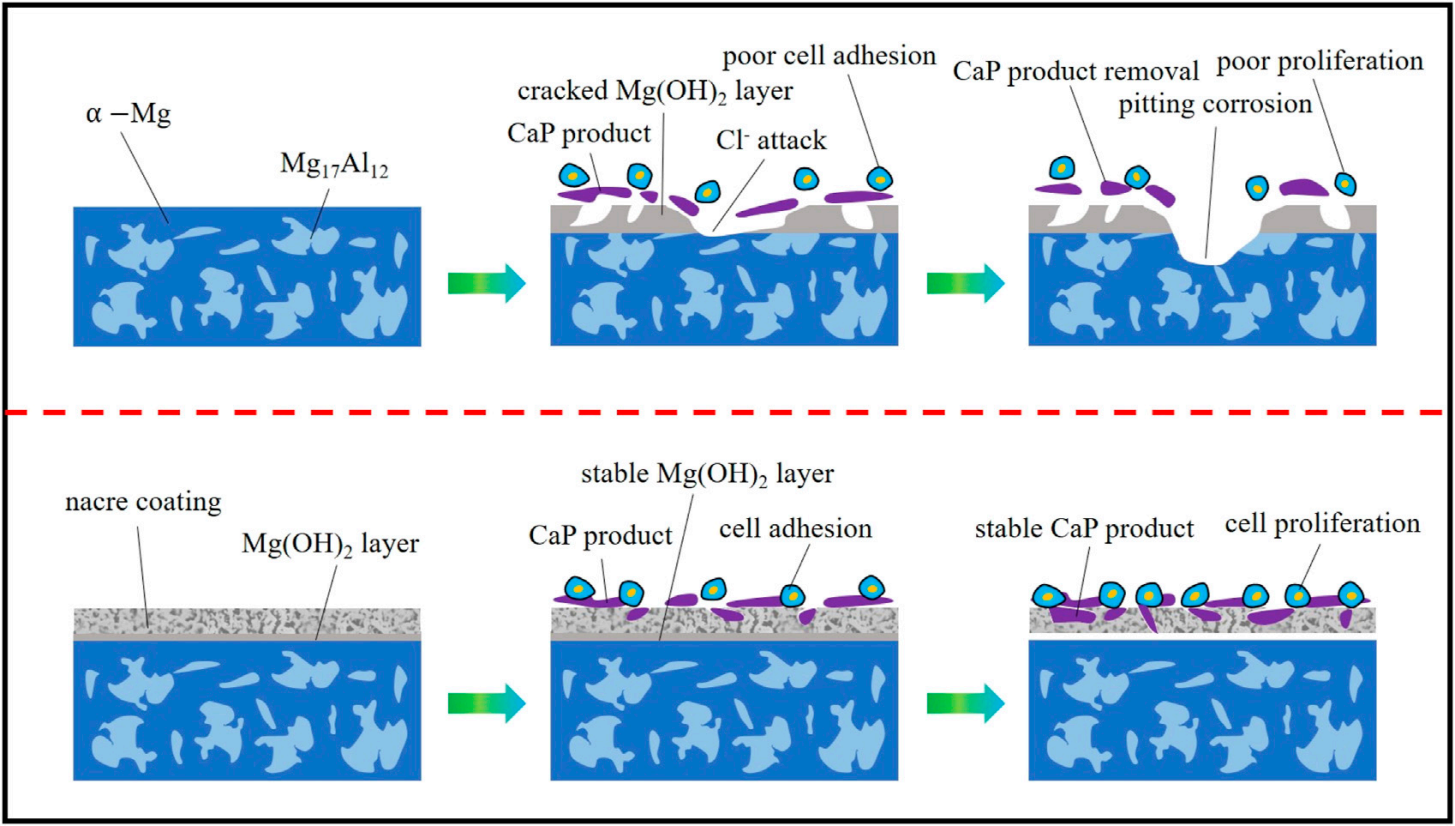
Schematic representation of degradation control on coated sample compared to bare AZ91D.

The schematic representation of degradation control on coated samples compared with bare AZ91D is shown in Figure 14. In the case of bare AZ91D, uncontrolled degradation reactions occur in the physiological environment. Soon after immersion, the Mg(OH)_2_-oxidized layer is generated on the surface. The oxidized layer has a low Pilling–Bedworth ratio; thus, excessive tensile stress or compressive stress is easy to produce, resulting in crack formation. Additionally, the Cl^−^present in human body fluids attacks the Mg(OH)_2_ layer to form soluble MgCl_2_ and causes severe pitting corrosion. In the case of coated samples, the acid pretreatment process combined with nacre coating greatly reduces the degradation rate of AZ91D alloys. As the main barrier to prevent the penetration of the medium, the nacre coating enhances the corrosion resistance of the AZ91D alloy in the physiological environment via physical and chemical protection. It can not only hinder the ionization of Mg^2+^ from the AZ91D substrate but also adsorb the Cl^−^present in human body fluids, thereby inhibiting the occurrence of pitting corrosion. Moreover, the Mg(OH)_2_ layer can perform as a second protective barrier for the substrate and maintain the stabilization of the interface, even though human body fluids can penetrate the substrate through the micropores of the nacre coating. In addition, the micropores in the nacre coating can supply CaP corrosion product particles with numerous available sites for nucleation and growth. Meanwhile, the high stability of the interface can facilitate more CaP products to fill the corrosion pores in the coating, endowing the coating with an ability of re-biomineralization. On the one hand, the nacre coating can delay the precipitation of harmful metal ions by inhibiting the rapid degradation of the AZ91D alloy substrate. On the other hand, the CaP component similar to human bone mineral in the coating and the protein, amino acid, and other active ingredients in pearl powder can induce the nucleation and growth of osteoblasts and promote the adhesion and proliferation of cells.

## Conclusion

1) The nacre coatings with high crystallinity can be successfully prepared on the surface of AZ91D alloy substrates via the hydrothermal method. The pH of the hydrothermal solution affects the composition and microstructure of the coating. The deposited coating is composed of DCPA with flower-like morphology after treatment at pH = 5.5. The composition of coatings is gradually transformed from DCPA to HA when the pH values increase to 8.5 and 11.5, and the microstructure of coatings changes into flocculent precipitates.
2) The nacre coatings can effectively decrease the degradation rate of AZ91D alloys in SBF. E_corr_, E_break_, and R_ct_ of bare AZ91D hydrothermally treated samples are lower than hydrothermally treated samples, and i_corr_ of coated samples considerably decreases. Moreover, the Ca and P contents on the surface of all coated samples increase after the electrochemical test, showing the ability of further biomineralization.
3) The biocompatibility of Mg alloys is efficaciously improved due to the CaP component, which is similar to the human bone mineral in coatings and the protein, amino acid, and other active ingredients in pearl powder.

## Data Availability

The original contributions presented in the study are included in the article/Supplementary Material, further inquiries can be directed to the corresponding author.
